# Risk factors for oral health in young, urban, Aboriginal and Torres Strait Islander children

**DOI:** 10.1111/adj.12662

**Published:** 2018-12-02

**Authors:** K Butten, NW Johnson, KK Hall, J Anderson, M Toombs, N King, KF O'Grady

**Affiliations:** ^1^ Queensland University of Technology Institute of Health & Biomedical Innovation Centre for Children's Health Research South Brisbane Queensland Australia; ^2^ Menzies Health Institute Queensland and School of Dentistry and Oral Health Griffith University Gold Coast Queensland Australia; ^3^ Dental Institute King's College London London UK; ^4^ Caboolture Community Medical Caboolture Queensland Australia; ^5^ Rural Clinical School The University of Queensland South Toowoomba Queensland Australia; ^6^ Faculty of Health Queensland University of Technology Kelvin Grove Queensland Australia; ^7^ Present address: Griffith University 170 Kessels Road Nathan Campus Qld 4111 Australia

**Keywords:** Aboriginal and Torres Strait Islander, children, dental caries, risk factors, urban

## Abstract

**Background:**

The caries process follows a strong social gradient which can commence in the first years of life**.** Yet data on young children remain limited. This study reports the potential risk factors and indicators in urban, Aboriginal and Torres Strait Islander children aged less than 5 and estimates the prevalence of caries.

**Methods:**

Demographic and risk factor and risk indicator data were collected at baseline in a cohort study of children attending a health clinic in north Brisbane. Dentulous children received a basic oral examination to explore the presence of decayed, missing and filled teeth (dmft). Descriptive analyses were performed. A backwards stepwise logistic regression model was performed to identify potential associations with dmft status.

**Results:**

In this study, 180 children enrolled: 111 children received the oral examination, of whom 14 (12.6%) (mean age 35 months) were estimated to have dmft >0. There was a high prevalence of socio‐economic, dietary and behavioural risk factors/indicators present for children. Due to the small sample size, planned regression was not performed.

**Conclusions:**

Overall, the prevalence of risk factors and risk indicators for caries in the study population is high. More culturally appropriate resources that support preventive care need to be invested before children are school aged.

**Abbreviations and acronyms**
ADG: Australian Dietary Guidelines
QCOHS: Queensland Child Oral Health Survey

## Background

Within Australia, dental caries is the most common chronic disease of childhood^1^ and Aboriginal and Torres Strait Islander (hereafter respectfully referred to as Indigenous) children experience a disproportionate amount of that burden.^2^ Despite being acknowledged as a priority population by the Australian Government's National Oral Health Plan^1^ since 2004, data on the oral health of Indigenous peoples are lacking, particularly for those living in urban areas and young children. Indeed, there is a lack of data nationally on the oral health of children aged less than 5 years.^3^, ^4^

It is important to address this gap as recent research suggests that a cariogenic environment may be established early on and preventive measures should take place in the first year of life before deciduous dentition commences.^3^ Caries are associated with a number of different risk factors and risk indicators. In this paper, we define a risk factor as an attribute in the direct causal chain of the disease (such as poor oral hygiene and high sugar diet), and a risk indicator as an attribute which is not of itself causal, but which has an indirect influence on the outcome (e.g. parenting practices) by influencing exposure to real risk.^4^, ^5^ Individual factors attributed to the increased risk of early childhood caries include: going to sleep with a bottle, regular exposure to sugar through food or drink, not visiting a dentist, and lack of preventive care i.e. brushing and fluoride exposure.^6^, ^7^ Socio‐demographic factors including low‐income, being a single‐parent, and low levels of parent education are also well‐established risk indicators for caries.^8^ Furthermore, it is hypothesized that cultural factors such as the impact of colonization and the transgenerational experience of dispossession may also be associated with caries risk within Indigenous populations.^9^ Historically, the oral health of Indigenous peoples was considered good, if not superior to non‐Indigenous peoples.^2^ However, it is well known that colonization and transgenerational inequities have negatively impacted the overall health of Indigenous people.^9^ Cultural factors such as family history (e.g. Stolen Generation), community involvement, connection to country and partaking in traditional cultural practises have not been studied in relation to oral health.

National data collections currently do not adequately capture Indigenous people and research has mostly taken place in rural and remote communities.^2^ Risk factors and indicators for different populations may vary, particularly between urban, rural, and remote populations. Thus, we aimed to determine the prevalence of known risk factors and risk indicators for caries in Indigenous children aged less than 5 years in an urban population and identify the characteristics of a child at risk for caries inclusive of demographic, behavioural, physiological, and cultural factors.

## Methods

The study was approved by the ethics committees of the Queensland Children's Health Services (HREC/12/QRCH/169), University of Queensland (2012001395) and Queensland University of Technology (13000000741). The study was registered with the Australia New Zealand Clinical Trials Registry (ACTRN 12614001214628). Cultural oversight was provided by an Indigenous Research Reference Group.

This paper reports on results from an opportunistic oral health investigation conducted during a prospective cohort study investigating acute respiratory illness in children aged less than 5 years. The full study protocol has been published.^10^

Recruitment and data collection were opportunistic and was conducted through an Aboriginal owned and operated primary health care clinic in Caboolture, a northern suburb of Brisbane, Queensland, between February 2013 and October 2015. Clients with children were approached in the waiting room by an Aboriginal research officer. Children were eligible for the study if they were aged less than 5 years, registered as a patient of the clinic, and the parent/carer was willing and able to consent and complete the study requirements. Children were excluded if the family was planning to move in the following 12 months. The study protocol was explained verbally and a plain language written statement was provided to potential participants. Signed consent was obtained.

A parent/carer questionnaire was used to collect data at enrolment. Information collected included demographic, cultural, economic, and clinical factors. Factors cited in the literature as established or potential risk factors and risk indicators of dental disease were also collected. These included both dietary and oral health behaviours of parent and child.

The oral examination was basic, limited by the child's age and acceptance of the examination to be conducted, and by the staffing and facilities of the organization. Infants and younger children were held in their parents arms with their head tilted back. Older children sat in a chair independently. A dental hygienist or the researcher who had been trained by the hygienist visually inspected the mouth and used a gloved hand and mirror when possible (younger children sometimes did not accept the use of the mirror) to look and feel for the number of teeth present and/or erupting and whether teeth had cavities present, were filled or were missing as a result of decay. Teeth were considered present if they had erupted through the gum and could be exposed to saliva, food, and microflora. Teeth were marked as having decay if cavitation to the dentine was visible to the naked eye. Plaque levels and non‐cavitated lesions were not counted. As there were only two examiners and the one taught the other, they were not calibrated. Decayed, missing and filled deciduous teeth (dmft) were noted on a data collection sheet based on the ‘dmft index’ criteria proposed by the World Health Organization.^11^

Descriptive analyses were performed for all children with proportions presented by children with and without deciduous teeth present. Further analyses were performed on data from children who had deciduous teeth present and had undergone a dental screen. Due to the small sample size, whether children with deciduous teeth present had any evidence of decayed, missing or filled teeth (dmft) was dichotomized as either ‘yes’ having dmft or ‘no’ not having any dmft; proportions were presented by dmft status, ‘dmft‐yes’ or ‘dmft‐no’. Chi‐squared and Fischer's exact (for variables with cell counts <5) statistics were used to look for differences in both the teeth present and not yet present groups and dmft ‘yes’ or ‘no’ groups. In the dmft status group (i.e. the 111 children who had teeth present and had a dental screen to determine if they had any dmft, we planned to enter factors that had a *P* value less of <0.2 into a backwards stepwise logistic regression model to identify potential associations with dmft status. All analyses were conducted using Stata 13.1 software (Stata Corp, College Station, TX, USA).

## Results

In this study, 403 children were screened and 200 children enrolled. Of those not enrolled, 72 (35.4%) refused, 43 (21.6%) were ineligible and 88 (43.3%) were not enrolled for other reasons, such as homelessness or because the child was not with a primary carer or legal guardian at the time. Of those enrolled, 180 identified as Indigenous and were eligible for this analysis. The median age was 18.4 months (interquartile range 7.7–34.3); 51% were male. The majority of children 138 (76.6%) had deciduous teeth present and of the 138, 111 received a dental examination. The characteristics of study children by deciduous teeth present or not present are described in Table 1.

**Table 1:** Child demographic and cultural characteristics

		All children N = 180 (%)	Deciduous teeth not present N = 42 (%)	Deciduous teeth present N = 138 (%)	*P* value*
Gender	Female	88 (48.9)	21 (50.0)	67 (48.5)	0.86
	Male	92 (51.1)	21 (50.0)	71 (51.4)	
Age group	<12 months	59 (32.7)	42 (100.0)	17 (12.3)	<0.01
	12 to <24 months	48 (26.6)	0 (0.0)	48 (34.7)	
	24 to <36 months	29 (16.1)	0 (0.0)	29 (21.0)	
	36 to <48 months	22 (12.2)	0 (0.0)	22 (15.9)	
	≥48 months	22 (12.2)	0 (0.0)	22 (15.9)	
Birth age	<37 weeks	34 (18.8)	9 (21.4)	25 (18.3)	0.66
	>37 weeks	144 (80.0)	33 (78.5)	111 (81.6)	
	Missing	2 (1.1)	0 (0.0)	2 (1.4)	
Birth weight	≤2500 g	31 (17.2)	8 (19.0)	23 (16.6)	0.72
	>2500 g	149 (82.7)	34 (80.9)	115 (83.3)	
Child has a chronic illness	No	162 (90.0)	39 (92.8)	123 (89.1)	0.57
	Yes	18 (10.0)	3 (7.1)	15 (10.8)	
Diagnosed respiratory illness in past 12 months	No	118 (65.5)	38 (90.4)	80 (57.9)	0.00
	Yes	61 (33.8)	4 (9.5)	57 (41.3)	
	Missing	1 (0.5)	0 (0.0)	1 (0.7)	
Toothache in past 7 days	No	177 (98.3)	42 (100.0)	135 (97.8)	1.00
	Yes	3 (1.6)	0 (0.0)	3 (2.1)	
Mother smoked during pregnancy	No	90 (50.0)	23 (54.7)	67 (48.5)	0.50
	Yes	89 (49.4)	19 (45.2)	70 (50.7)	
	Missing	1 (0.5)	0 (0.0)	1 (0.7)	
Mother consumed alcohol during pregnancy	No	147 (81.6)	33 (78.5)	114 (82.6)	0.49
	Yes	32 (17.7)	9 (21.4)	23 (16.6)	
	Missing	1 (0.5)	0 (0.0)	1 (0.7)	
Child was breastfed ever	No	49 (27.3)	11 (26.1)	38 (27.7)	0.84
	Yes	130 (72.1)	31 (73.8)	99 (72.2)	
	Missing	1 (0.5)	0 (0.0)	1 (0.7)	
Maternal age at birth	<25 years	93 (51.6)	19 (45.2)	74 (53.6)	0.15
	25–30 years	37 (20.5)	7 (16.6)	30 (21.7)	
	30+ years	50 (27.7)	16 (38.1)	34 (24.6)	
Paternal age at birth	<25 years	65 (36.1)	11 (26.1)	54 (39.1)	0.01
	25–30 years	47 (26.1)	7 (16.6)	40 (28.9)	
	30+ years	68 (37.7)	24 (57.1)	44 (31.8)	
Mother highest education	Tertiary	3 (1.6)	2 (4.7)	1 (0.7)	0.03
	Certificate/diploma	22 (12.2)	4 (9.5)	18 (13.0)	
	High school	85 (47.2)	26 (61.9)	59 (42.7)	
	Did not complete high school	68 (37.7)	10 (23.8)	58 (42.0)	
	Unknown/missing	2 (1.1)	0 (0.0)	2 (1.4)	
Father highest education	Tertiary	0 (0.0)	0 (0.0)	0 (0.0)	0.10
	Certificate/diploma	22 (12.2)	5 (11.9)	17 (12.3)	
	High school	52 (28.8)	18 (42.8)	34 (24.6)	
	Did not complete high school	81 (45.0)	15 (35.7)	66 (47.8)	
	Declined/unknown/missing	25 (13.8)	4 (9.5)	21 (15.2)	
Mother's employment	Employed	25 (85.5)	5 (11.9)	20 (14.4)	0.66
	Unemployed	154 (85.5)	37 (88.1)	117 (87.7)	
	Missing	1 (0.5)	0 (0.0)	0 (0.0)	
Father's employment	Employed	77 (42.7)	21 (50.0)	56 (40.5)	0.57
	Unemployed	77 (42.7)	18 (42.8)	59 (42.7)	
	Declined/unknown/missing	26 (14.4)	0 (0.0)	0 (0.0)	
Annual household income	$101 000 to $156 000	5 (2.7)	1 (2.3)	4 (2.9)	0.54
	$78 000 to $104 000	12 (6.6)	4 (9.5)	8 (5.8)	
	$52 000 to $78 000	25 (13.8)	7 (16.6)	18 (13.0)	
	$26 000 to $52 000	69 (38.3)	18 (42.8)	51 (36.9)	
	<$26 000	69 (38.3)	12 (28.5)	51 (41.3)	
Primary carer on welfare	No	18 (10.0)	5 (11.9)	13 (9.4)	0.63
	Yes	162 (90.0)	37 (88.1)	125 (90.5)	
Private health insurance	No	171 (95.0)	39 (92.8)	132 (95.6)	0.46
	Yes	9 (5.0)	3 (7.1)	6 (4.3)	
Care type at home	Both parents	100 (55.5)	27 (64.2)	73 (52.9)	0.35
	Single parent	69 (38.3)	14 (33.3)	55 (39.8)	
	Other	11 (6.11)	1 (2.3)	10 (7.2)	
Total number of people in	≤2	10 (5.5)	1 (2.3)	9 (6.5)	0.41
	3–4	81 (45.2)	16 (38.1)	65 (47.4)	
	5–6	69 (38.5)	19 (45.2)	50 (36.5)	
	7+	19 (10.6)	6 (14.2)	13 (9.4)	
	Missing	1 (0.5)	0 (0.0)	1 (0.7)	
Total number of people in bedroom with child	0	20 (11.1)	1 (2.3)	19 (13.7)	<0.01
	1	80 (44.4)	14 (33.3)	66 (47.8)	
	2	43 (23.8)	11 (26.1)	32 (23.1)	
	3+	36 (20.0)	16 (38.1)	20 (14.4)	
	Missing	1 (0.5)	0 (0.0)	1 (0.7)	
Indigenous status of father	Indigenous	118 (65.5)	24 (57.1)	94 (68.1)	0.30
	Non‐Indigenous	59 (32.7)	17 (40.4)	42 (30.4)	
	Unknown/missing	3 (1.6)	1 (2.3)	2 (1.4)	
Indigenous status of mother	Indigenous	121 (67.2)	28 (66.6)	93 (67.3)	0.93
	Non‐Indigenous	59 (32.7)	14 (33.3)	45 (32.6)	
Connection to homeland	No	93 (51.6)	21 (50.0)	72 (52.1)	0.94
	Yes	78 (43.3)	19 (45.2)	59 (42.7)	
	Unknown	9 (5.0)	2 (4.7)	7 (5.0)	
Maintain cultural connections at home	No	68 (37.7)	14 (33.3)	54 (39.1)	0.45
	Yes	110 (61.1)	28 (66.6)	82 (59.4)	
	Unknown	2 (1.1)	0 (0.0)	2 (1.4)	
Connection to community	No	39 (21.6)	8 (19.0)	31 (22.4)	0.92
	Yes	137 (76.1)	33 (78.5)	104 (75.3)	
	Unknown	4 (2.2)	1 (2.3)	3 (2.1)	
Family from stolen generation	No	42 (23.3)	9 (21.4)	33 (23.9)	0.72
	Yes	82 (45.5)	19 (45.2)	63 (45.6)	
	Unknown	56 (31.1)	14 (33.3)	42 (30.4)	

*Chi‐squared test for trend, Fischer's exact if cell size <5.

Demographic characteristics (Table 1) were similarly distributed between those children with and without deciduous teeth present and a number of demographic‐associated risk indicators for caries were present: 76.6% of families had an annual household income of <$52 000, 51.6% of mothers were aged less than 25 at the time of their child's birth, 37.7% of mothers and 45.0% of fathers had not completed high school (Table 1).

Of the household and cultural factors assessed (Table 1) 45.5% of carers reported having family from the Stolen Generation, 23.3% reported that they did not and 31.1% did not know if their family was from the Stolen Generation. The majority, 76.1% said that they identified with an Indigenous community, 43.3% had a connection with their traditional homeland and 61.1% maintained cultural connections at home (Table 1).

Oral health behaviours for the child and carer are presented in Table 2 and indicate that, of the children that had deciduous teeth present, 81.1% of children brushed their teeth daily, 89.8% of children had their own toothbrush, and 86.6% of children used toothpaste (Table 2). Whether or not, the toothpaste contained fluoride was not recorded, but in the geographical area, the vast majority of toothpaste purchased is fluoridated, as is the council water supply since 2010. The majority (75.5%) of carers had self‐reported decayed, missed or filled teeth at the time of the study, and 48.0% indicated that it had been years since they had been to a dentist (Table 2).

**Table 2:** Oral health behaviours of child and carer

		All children N = 180 (%)	Deciduous teeth not present N = 42 (%)	Deciduous teeth present N = 138 (%)	*P* value*
Exclusively breastfed	No	154 (86.0)	22 (52.3)	132 (96.3)	0.86
	Yes	24 (13.4)	20 (47.6)	4 (2.9)	
	Unknown/missing	2 (1.4)	0 (0.0)	2 (1.4)	
Child had bottle at bedtime ever	No	89 (49.4)	32 (76.1)	57 (41.3)	<0.01
	Yes	82 (45.5)	7 (16.6)	75 (54.3)	
	Unknown/missing	9 (5.0)	3 (7.1)	6 (4.3)	
Child sucked thumb ever	No	154 (85.5)	35 (83.3)	119 (86.2)	1.00
	Yes	18 (10.0)	4 (9.5)	14 (10.1)	
	Unknown/missing	8 (4.4)	3 (7.1)	5 (3.6)	
Child used pacifier ever	No	93 (54.6)	23 (54.7)	70 (50.7)	0.46
	Yes	78 (43.3)	15 (35.7)	63 (45.6)	
	Unknown/missing	9 (5.0)	4 (9.5)	5 (3.6)	
Pacifier cleaned in carer mouth ever	No	141 (78.3)	30 (71.4)	111 (80.4)	0.46
	Yes	29 (16.1)	8 (19.0)	21 (15.2)	
	Unknown/missing/NA†	10 (5.5)	4 (9.5)	6 (4.3)	
Pacifier dipped in sweet ever	No	155 (86.1)	36 (85.7)	119 (96.2)	0.73
	Yes	14 (7.7)	2 (4.7)	12 (8.7)	
	Unknown/missing/NA	11 (6.1)	4 (9.5)	7 (5.0)	
Are child's teeth brushed	No	7 (3.8)	1 (2.3)	6 (4.3)	<0.01
	Yes	127 (70.5)	2 (4.7)	125 (90.5)	
	Not applicable	39 (21.6)	37 (88.1)	2 (1.4)	
	Unknown/missing	7 (3.8)	2 (4.7)	5 (3.5)	
Child uses toothpaste	No	6 (3.3)	3 (7.1)	3 (2.1)	<0.01
	Yes	120 (66.6)	0 (0.0)	120 (86.6)	
	Unknown/missing/NA	54 (29.8)	39 (92.8)	15 (10.8)	
Child has own toothbrush	No	2 (1.1)	1 (2.3)	1 (0.7)	0.04
	Yes	126 (70.0)	2 (4.7)	124 (89.8)	
	Unknown/missing/NA	52 (28.7)	39 (92.8)	13 (9.2)	
Frequency of brushing	>2 per day	9 (5.0)	0 (0.0)	9 (6.5)	<0.01
	1–2 per day	104 (57.7)	1 (2.3)	103 (74.6)	
	<1 per day	7 (3.8)	1 (2.3)	6 (4.3)	
	None	5 (2.7)	3 (7.1)	2 (1.4)	
	Unknown/missing/NA	55 (30.0)	37 (88.0)	18 (13.0)	
Child has been to dentist	No	157 (87.2)	39 (92.8)	118 (85.5)	0.07
	Yes	11 (6.1)	0 (0.0)	11 (7.9)	
	Unknown/missing	12 (6.6)	3 (7.1)	9 (6.5)	
Child likes dentist	No	14 (7.7)	0 (0.0)	14 (10.1)	N/A
	Yes	12 (6.6)	0 (0.0)	12 (8.7)	
	Unknown/missing/NA	154 (85.5)	42 (100.0)	112 (81.1)	
Last dental visit for carer	Months ago	23 (12.8)	5 (11.9)	18 (13.1)	0.57
	Years ago	86 (48.0)	18 (42.8)	68 (49.6)	
	Unknown	47 (26.6)	12 (28.5)	35 (25.5)	
	Never	3 (1.6)	2 (4.7)	1 (0.7)	
	Missing	21 (11.6)	5 (11.9)	16 (11.5)	
Carer likes dentist	No	68 (37.7)	16 (38.1)	52 (37.6)	0.71
	Yes	97 (53.8)	22 (52.3)	75 (54.3)	
	Unknown/missing	12 (8.3)	4 (9.4)	11 (7.9)	
Carer has DMFT	No	36 (20.0)	4 (9.5)	32 (23.1)	0.09
	Yes	136 (75.5)	35 (83.3)	101 (73.1)	
	Unknown/missing	8 (4.4)	3 (7.1)	5 (3.6)	
Carer perception of fluoride	Good	59 (32.7)	14 (33.3)	45 (32.6)	0.37
	Neither good nor bad	63 (35.0)	16 (38.1)	47 (34.0)	
	Bad	17 (9.4)	2 (4.7)	15 (10.8)	
	Don't know what fluoride is/unknown	33 (18.3)	6 (14.2)	27 (19.5)	
Carer has received education on dental health for child	No	95 (52.7)	25 (59.5)	70 (50.7)	0.94
	Yes	68 (37.7)	13 (30.9)	55 (39.8)	
	Unknown/missing	17 (9.4)	4 (9.5)	13 (9.3)	

*Chi squared test for trend excluding unknown/missing, Fischer's exact if cell size <5.

†Not applicable.

Dietary factors are displayed in Table 3. Beverages are described on a daily basis via bottle or cup and food products are described on a weekly basis, as labelled in Table 3. Only small numbers of children were having sweetened beverages in their bottles; 5% of all children were having juice in their bottle 1–3 times per day. However, the percentage increased (21.1%) for children having juice by way of a cup. Table 3 also reports on food items consumed per week. The majority of children who had yet to have deciduous teeth erupt were not eating foods included in the study, yet of those that had teeth, 32.6% were having hot chips (‘french fries’) 1–3 times per week and 25.3% were having confectionary once or more per week.

**Table 3:** Dietary variables

	Serving	All children N = 180 (%)	Deciduous teeth not present N = 42 (%)	Deciduous teeth present N = 138 (%)	P value*
Beverages in bottle per day					
Cordial ‘fruit drink concentrate’	None	166 (92.2)	19 (45.2)	37 (26.8)	0.17
	<1 per day	3 (1.6)	0 (0.0)	3 (2.1)	
	1–3 per day	2 (1.1)	0 (0.0)	2 (1.4)	
	>3 per day	2 (1.1)	0 (0.0)	2 (1.4)	
	Unknown/missing	7 (3.8)	2 (4.7)	5 (3.6)	
Carbonated drink	None	171 (95.0)	40 (95.2)	131 (94.9)	0.46
	<1 per day	1 (0.5)	0 (0.0)	1 (0.7)	
	1–3 per day	1 (0.5)	0 (0.0)	1 (0.7)	
	>3 per day	0 (0.0)	0 (0.0)	0 (0.0)	
	Unknown/missing	7 (3.8)	2 (4.7)	5 (3.6)	
Flavoured milk	None	168 (93.3)	40 (95.2)	128 (92.7)	0.04
	<1 per day	1 (0.5)	0 (0.0)	1 (0.7)	
	1–3 per day	2 (1.1)	0 (0.0)	2 (1.4)	
	>3 per day	1 (0.5)	0 (0.0)	1 (0.7)	
	Unknown/missing	8 (4.4)	2 (4.7)	6 (4.3)	
Fruit juice	None	156 (86.6)	36 (85.7)	120 (86.9)	0.99
	<1 per day	8 (4.4)	2 (4.7)	6 (4.3)	
	1–3 per day	9 (5.0)	2 (4.7)	7 (5.0)	
	>3 per day	0 (0.0)	0 (0.0)	0 (0.0)	
	Unknown/missing	7 (3.8)	2 (4.7)	5 (3.6)	
Plain milk	None	111 (61.6)	21 (50.0)	90 (65.2)	0.04
	<1 per day	0 (0.0)	0 (0.0)	0 (0.0)	
	1–3 per day	8 (4.4)	0 (0.0)	8 (5.8)	
	>3 per day	54 (30.0)	19 (45.2)	35 (25.3)	
	Unknown/missing	7 (3.8)	2 (4.7)	5 (3.6)	
Water	None	128 (71.1)	25 (59.5)	103 (74.6)	0.02
	<1 per day	0 (0.0)	0 (0.0)	0 (0.0)	
	1–3 per day	5 (2.7)	0 (0.0)	5 (3.6)	
	>3 per day	39 (21.6)	15 (35.7)	24 (17.3)	
	Unknown/missing	8 (4.4)	2 (4.7)	6 (4.3)	
Beverages in cup per day					
Cordial ‘fruit drink concentrate’	None	118(65.9)	40(95.2)	78(56.9)	<0.01
	<1 per day	36(20.1)	0(0.0)	36(26.2)	
	1–3 per day	10(5.5)	0(0.0)	10(7.3)	
	>3 per day	7(3.9)	0(0.0)	7(5.1)	
	Unknown/missing	8(4.4)	2(4.7)	6(4.3)	
Carbonated drink	None	120(66.6)	39(92.8)	81(58.7)	<0.01
	<1 per day	40(22.2)	1(2.3)	39(28.2)	
	1–3 per day	10(5.5)	0(0.0)	10(5.5)	
	>3 per day	2(1.1)	0(0.0)	2(1.4)	
	Unknown/missing	8(4.4)	2(4.7)	6(4.3)	
Flavoured milk	None	128(71.1)	40(95.2)	88(63.7)	<0.01
	<1 per day	33(18.3)	0(0.0)	33(23.9)	
	1–3 per day	9(5.0)	0(0.0)	9(6.5)	
	>3 per day	2(1.1)	0(0.0)	2(1.4)	
	Unknown/missing	8(4.4)	2(4.7)	6(4.3)	
Fruit juice	None	84(46.6)	37(88.1)	47(34.0)	<0.01
	<1 per day	44(24.4)	2(4.7)	42(30.4)	
	1–3 per day	38(21.1)	1(2.3)	37(26.8)	
	>3 per day	6(3.3)	0(0.0)	6(4.3)	
	Unknown/missing	8(4.4)	2(4.7)	6(4.3)	
Plain milk	None	43(23.8)	27(64.2)	16(11.5)	<0.01
	<1 per day	6(3.3)	0(0.0)	6(4.3)	
	1–3 per day	35(19.4)	1(2.3)	34(24.6)	
	>3 per day	87(48.3)	12(28.5)	7(5.0)	
	Unknown	9(5.0)	2(4.7)	7(5.0)	
Water	None	35(19.4)	27(64.2)	8(5.8)	<0.01
	<1 per day	1(0.5)	1(2.3)	0(0.0)	
	1–3 per day	23(12.7)	1(2.3)	22(15.9)	
	>3 per day	113(62.7)	11(26.1)	102(73.9)	
	Unknown/missing	8(4.4)	2(4.7)	6(4.3)	
Food items per week					
Cereal	None	123 (68.3)	39 (92.8)	84 (60.8)	<0.01
	<1 per week	34 (18.8)	1 (2.3)	33 (23.9)	
	1–3 per week	6 (3.3)	0 (0.0)	6 (4.3)	
	>3 per week	7 (3.8)	0 (0.0)	7 (5.0)	
	Unknown/missing	10 (5.5)	2 (4.7)	8 (5.8)	
Chocolate	None	81 (45.0)	38 (90.4)	43 (31.1)	<0.01
	<1 per week	60 (33.3)	2 (4.7)	58 (42.0)	
	1–3 per week	18 (10.0)	0 (0.0)	18 (13.0)	
	>3 per week	12 (6.6)	0 (0.0)	12 (8.7)	
	Unknown/missing	9 (5.0)	2 (4.7)	7 (5.0)	
Fresh fruit	None	48 (26.6)	35 (83.3)	13 (9.4)	<0.01
	<1 per week	5 (2.7)	1 (2.3)	4 (2.9)	
	1–3 per week	38 (21.1)	4 (9.5)	34 (24.6)	
	>3 per week	80(44.4)	0(0.0)	80(57.9)	
	Unknown/missing	9(5.0)	2(4.7)	7(5.0)	
Honey	None	118(65.5)	40 (95.2)	78 (56.5)	<0.01
	<1 per week	39(21.6)	0(0.0)	39(28.2)	
	1–3 per week	6(3.3)	0(0.0)	6(4.3)	
	>3 per week	7(3.8)	0(0.0)	7(5.0)	
	Unknown/missing	10(5.5)	2(4.7)	8(5.8)	
Hot chips ‘french fries’	None	69(38.3)	38(90.4)	31 (22.4)	<0.01
	<1 per week	46 (25.5)	1 (2.3)	45 (32.6)	
	1–3 per week	46 (25.5)	1 (2.3)	45 (32.6)	
	>3 per week	9 (5.0)	0 (0.0)	9 (6.5)	
	Unknown/missing	10 (5.5)	2 (4.7)	8 (5.8)	
Jam/fruit conserve	None	125 (69.4)	40 (95.2)	85 (61.5)	<0.01
	<1 per week	34 (18.8)	0 (0.0)	34 (24.6)	
	1–3 per week	6 (3.3)	0 (0.0)	6 (4.3)	
	>3 per week	5 (2.7)	0 (0.0)	5 (3.6)	
	Unknown/missing	10 (5.5)	2 (4.7)	8 (5.8)	
Confectionary	None	79 (43.8)	38 (90.4)	41 (29.7)	<0.01
	<1 per week	55 (30.5)	2 (4.7)	53 (38.4)	
	1–3 per week	22 (12.2)	0 (0.0)	22 (15.9)	
	>3 per week	13 (7.2)	0 (0.0)	13 (9.4)	
	Unknown/missing	11 (6.1)	2 (4.7)	9 (6.5)	
Potato chips ‘crisps’	None	93 (51.6)	39 (92.8)	54 (39.1)	<0.01
	<1 per week	49 (27.2)	1 (2.3)	48 (34.7)	
	1–3 per week	15 (8.3)	0 (0.0)	15 (10.8)	
	>3 per week	13 (7.2)	0 (0.0)	13 (9.4)	
	Unknown/missing	10 (5.5)	2 (4.7)	8 (5.8)	
Tinned fruit	None	118 (65.5)	38 (90.4)	80 (57.9)	<0.01
	<1 per week	33 (18.3)	1 (2.3)	32 (23.1)	
	1–3 per week	14 (7.7)	1 (2.3)	13 (9.4)	
	>3 per week	4 (2.2)	0 (0.0)	4 (2.9)	
	Unknown/missing	11 (6.1)	2 (4.7)	9 (6.5)	

*Chi‐squared test for trend excluding unknown/missing, Fischer's exact if cell size <5.

Of the 138 children who had deciduous teeth present, 111 children received a dental examination. The majority 97 (87.3%) had no observed dmft as a result of caries, compared with 14 (12.6%) children who did have dmft from caries i.e. dmft >0. The mean dmft was 3.0 teeth (standard deviation (SD) 1.7). The mean age for children with dmft experience was 35 months. While there were a number of factors that were identified on univariate analysis for inclusion in regression models, the small sample, the small number of children with dmft, missing data for some variables, and the small values in some cells, meant that a statistically valid model could not be constructed, particularly when accounting for the effect of age. Factors eligible for inclusion are available for viewing in supplementary tables online.

## Discussion

Understanding the current oral health environment of young, urban Indigenous children is necessary to inform policy and future programmes. This study is one of few Australian studies^4^, ^12^, ^13^, ^14^ to report the characteristics of young children in relation to their oral health and is believed to be the first in over a decade to report on the prevalence of risk indicators and risk factors for caries in Indigenous children aged <5 living in urban Australia.^2^

We found that the prevalence of caries (12.6%), as well as the mean dmft (3.0 teeth, SD 1.7) was higher than has been reported in other studies^12^, ^13^, ^14^ for other Australian children of a similar age (mean age 35 months in our study). A longitudinal case–control study (2008)^14^ of caries development from birth to 36 months conducted in a comparable socio‐economic area to our study (Logan, south of Brisbane) reported that 9% of the 552 children aged 36 months had dental caries (i.e. cavitated lesions). The mean dmft was 2.3 teeth (SD 1.5).^14^ It is speculated that the contact by an oral health therapist every 6 months either by telephone or in a home visit to children during the Logan study may have contributed to the lower prevalence of dmft compared to our study population.

Overall, the prevalence of risk factors and risk indicators for caries in the study population is high. Poverty is one of the greatest influencers of oral health^1^ and the study population is considerably economically disadvantaged; 42.7% of fathers and 85.5% of mothers were unemployed and 76.6% of households had an annual income of <$52 000. The Australian National Dental Telephone Interview Survey 2010^15^ reported that cost was a major barrier to people accessing oral health services and those in the lowest income level were the least likely to receive preventive oral health care.^15^ In this study, 48.0% of carers had reportedly not visited a dentist in ‘years’ and 26.0% answered ‘unknown’ when asked when they last saw a dentist. A qualitative study found that apprehension towards dentists by Indigenous Australians was attributed to feeling judged by the dentist and having their health behaviours questioned.^16^ In our study, 75.5% of parents/carers reported that at the time of the study, they had decayed, missing or filled teeth themselves, and 37.7% indicated that they did not like going to the dentist. Fifty‐nine of the 75 respondents suggested that they were frightened; others indicated it was a mixture of fear, shame and/or embarrassment. The need for culturally safe care within the Australian health care system is well established.^16^ Programmes that are culturally safe and support parents to attend the dentist can potentially influence children's attendance; with the relationship between a carer's oral health behaviours and a child's caries risk having been demonstrated previously.^17^

In our study, children's attendance to the dentist was higher (18.1%) than has been found for other Indigenous children of a similar age; 8% of children aged 30 months in Sydney, Australia (between 2005 and 2007) and 13.3% of children <36 months in the Queensland Child Oral Health Survey 2010–2011 (QCOHS).^18^ It was slightly lower compared with non‐Indigenous children (20.5%) as reported in the QCOHS.^18^ Like other studies,^15^, ^19^ the QCOHS indicated that children with the lowest family income were the least likely to attend the dentist.^18^ It is possible that the change to the Child Dental Benefits Schedule in Queensland, which extended the eligibility to children aged 2–4, when previously it provided for school‐aged children only, could have improved attendance in our study population.^20^ However, as this change in policy came half‐way through our data collection timeline, we can only speculate the influence. Other studies have reported that cultural connections and a strong Indigenous identity is protective for Indigenous people's health, particularly for Indigenous children.^21^, ^22^ Despite over half of parents and carers, suggesting they had received no education on caring for their child's oral health, 90.5% of children with deciduous teeth present had their teeth brushed, 74.6% of those children brushing 1–2 times per day. Three quarters of participants in our study indicated they had a connection to an Indigenous community and 61.1% maintained cultural practices at home. The prevalence of reported cultural connection within our study community could be a protective factor although this would need to be explored further in larger studies.

Dietary habits have considerable influence on the oral health of young children, particularly night‐time feeding with bottles and the regular consumption of cariogenic food and beverages.^3^, ^4^ In our study, it was encouraging to see that nearly half of children never had a bottle at bedtime and very few were having sugar‐sweetened beverages in their bottles on a daily basis. However, there was a marked increase in the consumption of both cariogenic food and beverages once children had one or more deciduous teeth present and were drinking from a cup. This likely reflects the increased age of the child. Yet the consumption of ‘discretionary foods’ as described by the Australian Dietary Guidelines (ADG)^23^ is not recommended for young children. The ADG advises that discretionary foods are “not an essential or necessary part of healthy dietary patterns” and children <12 months should not be consuming them at all and older children should have them “only sometimes and in small amounts”.^23^ In univariate analyses, a number of foods/drinks were associated with dmft, as reported in other studies.^3^, ^4^, ^23^ However, we could not examine them in regression analyses; larger studies are required to investigate these associations further. A qualitative study of parents of young children found that parents were more likely to give sugary beverages to children as they aged to appease children's preference and temperament.^24^ There was also the notion that it was more acceptable to give older children sugary beverages compared with infants.^24^ Programmes that support parent's self‐efficacy to continue with the healthy choices they are making for their infant could potentially be beneficial. As has been shown in our study, parents appear to be supporting positive oral health behaviours, but there is room for improvement, especially as children age.

There are limitations to our study, including the method of oral screening. Due to the age of children and facility resources, only basic visual examinations were conducted and obvious decay recorded. Some argue that cavitated lesions, as documented for the dmft index, should not be the only marker for disease and pre‐cavitated lesions should also be recorded as they contribute to disease trajectory. Given the insensitivity of the dmft measurement index, researchers should consider that the burden of disease is likely higher in a study population.^13^ In this study, due to small numbers, we dichotomized the dmft status. The Australian Research Centre for Population Oral Health^25^ posits that by looking at mean dmft scores, and in our case, a yes or no status, we are potentially overlooking the number of children that are carrying a disproportionate amount of the disease.^25^ Also, impacted by the sample size was our ability to achieve a stable regression model. Despite a number of factors approaching or reaching significance in univariate analysis, a sound model could not be completed because of the small sample size, the small proportion with dmft, and some missing data. Other limitations for the study include its cross‐sectional nature, the self‐reporting of dietary data, and that all data were from a single centre.

Our study indicates that parents and carers of young, urban Indigenous children are engaged in supporting their child's oral health at home. However, there is also a high prevalence of several known risk factors and risk indicators for dental disease. Currently, many research studies and preventive programmes take place in school‐aged children. This study, and the few others investigating oral health of young children, indicate that more resources need to be invested before children are school‐aged, particularly culturally appropriate services that will increase healthy life styles amongst families, and the uptake of dental care in this population. Such dental care would need to be both affordable and appropriate, and to support parenting as argued recently by Kumar *et al*.^26^ and the authors of the La Cascada Declaration.^27^

## Disclosure

None declared.

## Supporting information


**Table S1.** Demographic and behavioural factors eligible for inclusion into a regression model.
**Table S2.** Dietary factors eligible for inclusion into a regression model.
