# Supplementary material for: Risk factors for oral health in young, urban, Aboriginal and Torres Strait Islander children
Source: Aust Dent J. 2018 Dec 2;64(1):72–81. doi: 10.1111/adj.12662 (PMC6392135; doi:10.1111/adj.12662)
Supplement: Supplementary file 1 — Table S1. Demographic and behavioural factors eligible for inclusion into a regression model. Table S2. Dietary factors eligible for inclusion into a regression model. [file ADJ-64-72-s001.docx]

**Table 1. Demographic and behavioural factors eligible for inclusion into a regression model**

|  | | All Screened N=111 (%) | Dmft ‘no’  N=97(%) | Dmft ‘yes’  N=14 (%) | P Value* |
| --- | --- | --- | --- | --- | --- |
| Gender | Female | 56 (50.4) | 50 (51.5) | 6 (42.85) | 0.54 |
|  | Male | 55 (49.5) | 47 (48.4) | 8 (57.1) |  |
| Age group | < 12 months | 14 (12.6) | 13 (13.4) | 1 (7.1) | 0.05 |
|  | 12 - <24 months | 36 (32.4) | 35 (36.0) | 1 (7.1) |  |
|  | 24 - <36 months | 26 (23.4) | 22 (22.6) | 4 (28.5) |  |
|  | 36 - <48 months | 18 (16.2) | 12 (12.3) | 6 (42.8) |  |
|  | >= 48months | 17 (15.3) | 15 (15.4) | 2 (14.2) |  |
| Total number of people  in house | <=2 | 7 (6.3) | 7 (7.2) | 0 (0.0) | 0.09 |
|  | 3-4 | 53 (47.7) | 49 (50.5) | 4 (28.5) |  |
|  | 5-6 | 44 (39.6) | 34 (35.0) | 10 (71.4) |  |
|  | 7+ | 7 (6.3) | 7 (7.2) | 0 (0.0) |  |
| Total number of people in bedroom with child | 0 | 16 (14.4) | 16 (16.4) | 0 (0.0) | 0.17 |
|  | 1 | 55 (49.5) | 49 (50.5) | 6 (42.8) |  |
|  | 2 | 28 (25.2) | 22 (22.6) | 6 (42.8) |  |
|  | 3+ | 12 (10.8) | 10 (10.3) | 2 (14.2) |  |
| Indigenous status of mother | Indigenous | 73 (65.7) | 66 (68.0) | 7 (50.0) | 0.18 |
|  | Non-Indigenous | 38 (34.2) | 31 (31.9) | 7 (50.0) |  |
| Paternal age at birth | <25 years | 42 (37.8) | 40 (41.2) | 2 (14.2) | 0.12 |
|  | 25-30 years | 33 (29.7) | 28 (28.8) | 5 (35.7) |  |
|  | 30+ years | 36 (32.4) | 29 (29.9) | 7 (50.0) |  |
| Mother highest education | Tertiary | 1 (0.9) | 0 (0.0) | 1 (7.1) | 0.07 |
|  | Certificate/diploma | 13 (11.7) | 13 (13.4) | 0 (0.0) |  |
|  | High school | 50 (45.0) | 42 (43.3) | 8 (57.1) |  |
|  | Did not complete high school | 46 (41.4) | 41 (42.2) | 5 (35.7) |  |
|  | Unknown/missing | 1 (0.9) | 1 (1.0) | 0 (0.0) |  |
| Frequency of brushing | > 2 per day | 5 (4.5) | 5 (5.1) | 0 (0.0) | 0.19 |
|  | 1 to 2 per day | 89 (80.1) | 79 (81.4) | 10 (71.4) |  |
|  | <1 per day | 4 (3.6) | 3 (3.0) | 1 (7.1) |  |
|  | None | 2 (1.8) | 1 (1.0) | 1 (7.1) |  |
|  | Unknown/missing/NA | 11 (9.9) | 9 (9.2) | 2 (14.2) |  |
| Child has been to dentist | No | 100 (90.0) | 90 (92.7) | 10 (71.4) | 0.15 |
|  | Yes | 9 (8.1) | 5 (5.1) | 4 (28.5) |  |
|  | Unknown/missing | 2 (1.8) | 2 (2.0) | 0 (0.0) |  |
| Carer has received education on dental health for child | No | 57 (51.3) | 51 (52.5) | 6 (42.8) | 0.05 |
|  | Yes | 49 (44.1) | 43 (44.3) | 6 (42.8) |  |
|  | Unknown/missing | 5 (4.5) | 3 (3.0) | 2 (14.2) |  |
| * Chi2 test for trend excluding unknown/missing, Fischer’s exact if cell size <5 | | | | | |

**Table 2. Dietary factors eligible for inclusion into a regression model**

|  | | All Screened N=111 (%) | Dmft ‘no’  N=97(%) | Dmft ‘yes’  N=14 (%) | P Value* |
| --- | --- | --- | --- | --- | --- |
| Water in bottle | None | 88 (79.2) | 74 (76.2) | 14 (100.0) | 0.18 |
|  | <1 per day | 0 (0.0) | 0 (0.0) | 0 (0.0) |  |
|  | 1 to 3 per day | 4 (3.6) | 4 (4.1) | 0 (0.0) |  |
|  | >3 per day | 18 (16.2) | 18 (18.5) | 0 (0.0) |  |
|  | Unknown/missing | 1 (0.9) | 1 (1.0) | 0 (0.0) |  |
| Carbonated drink in a cup | None | 70 (63.0) | 65 (67.0 | 5 (35.7) | 0.01 |
|  | <1 per day | 33 (29.7) | 27 (27.8) | 6 (42.8) |  |
|  | 1 to 3per day | 6 (5.4) | 4 (4.1) | 2 (14.2) |  |
|  | >3 per day | 1 (0.9) | 0 (0.0) | 1 (7.1) |  |
|  | Unknown/missing | 1 (0.9) | 1 (1.0) | 0 (0.0) |  |
| Flavoured milk | None | 74 (66.6) | 67 (69.0) | 7 (50.0) | 0.04 |
|  | <1 per day | 29 (26.1) | 25 (25.7) | 4 (28.5) |  |
|  | 1 to 3 per day | 6 (5.4) | 4 (4.1) | 2 (14.2) |  |
|  | >3 per day | 1 (0.9) | 0 (0.0) | 1 (7.1) |  |
|  | Unknown/missing | 1 (0.9) | 1 (1.0) | 0 (0.0) |  |
| Cereal | None | 71 (63.9) | 65 (67.0) | 6 (42.8) | 0.08 |
|  | <1 per week | 31 (27.9) | 23 (23.7) | 8 (57.1) |  |
|  | 1 to 3 per week | 4 (3.6) | 4 (4.1) | 0 (0.0) |  |
|  | >3 per week | 5 (4.5) | 5 (5.1) | 0 (0.0) |  |
| Potato chips ‘crisps’ | None | 48 (43.2) | 45 (46.3) | 3 (21.4) | 0.16 |
|  | <1 per week | 43 (38.7) | 36 (37.1) | 7 (50.0) |  |
|  | 1 to 3 per week | 13 (11.7) | 11 (11.3) | 2 (14.2) |  |
|  | >3 per week | 7 (6.3) | 5 (5.1) | 2 (14.2) |  |
| Fresh fruit | None | 12 (10.8) | 12 (12.3) | 0 (0.0) | 0.20 |
|  | <1 per week | 3 (2.7) | 3 (3.0) | 0 (0.0) |  |
|  | 1 to 3 per week | 31 (27.9) | 24 (24.7) | 7 (50.0) |  |
|  | >3 per week | 65 (58.5) | 58 (59.7) | 7 (50.0) |  |
| * Chi2 test for trend, Fischer’s exact if cell size <5 | | | | | |
